# An Experimental Exploration of the Effects of Exposure to Images of Nature on Rumination

**DOI:** 10.3390/ijerph15020300

**Published:** 2018-02-09

**Authors:** Sarah Elizabeth Golding, Birgitta Gatersleben, Mark Cropley

**Affiliations:** School of Psychology, Faculty of Health & Medical Sciences, University of Surrey, Guildford GU2 7XH, UK; b.gatersleben@surrey.ac.uk (B.G.); mark.cropley@surrey.ac.uk (M.C.)

**Keywords:** psychological restoration, natural environments, urban environments, restorative environments, rumination, mood

## Abstract

Exposure to natural environments has been shown to have beneficial effects on mood. Rumination is a thinking style associated with negative mood, and sometimes depression, and is characterized by repetitive, intrusive thoughts, often with a negative emotional element. This study investigated whether exposure to nature, operationalized using photographs presented as a slideshow, could aid reduction in levels of state rumination. An experimental, within-between (Time x Condition) participant design was used; participants (*n* = 58) undertook a presentation task designed to induce rumination and influence mood. Participants were then randomly allocated to either: watch a slideshow of a natural environment, watch a slideshow of an urban environment, or wait patiently with no distractions. Data were collected at baseline, after the presentation, and after the slideshow. Environmental exposure had no effect on levels of rumination or negative mood, but did have a significant effect on levels of positive mood, ‘being away’, and ‘fascination’. Positive mood declined in those who saw the urban slideshow, but remained the same in those who saw the nature slideshow, whilst levels of being away and fascination were highest in those who saw the nature slideshow. This study extends previous restorative environment research by exploring the effects of nature on rumination.

## 1. Introduction

Evidence suggests that exposure to natural environments, and images of natural environments, can have beneficial effects on mood and help people recover from mental fatigue and stress [1,2,3]. Rumination is a style of thinking that is associated with low mood and stressful stimuli. The purpose of this study was therefore to explore whether exposure to nature, in the form of a photographic slideshow, might also be beneficial for ruminative thinking.

### 1.1. Rumination

Rumination is a cognitive process characterized by repetitive, intrusive thoughts, often with a negative emotional element [4]. It is a form of self-focused attention and can be conceptualized as a response-style to stressors and negative life events [5,6]. Rumination involves dwelling on past events, whereas worry tends to be future-focused [4].

Repetitive negative thoughts are a common feature of mood disorders and rumination has been associated with poorer mental health, particularly depression and anxiety [7,8,9]. Rumination is not just of concern for clinical populations, however, as it is also associated with perceived levels of stress, and may interfere with recovery from work [10,11]. Rumination is consistently associated with higher levels of negative mood; a meta-analysis found this association was represented by large effect sizes in both correlational and experimental studies [7]. 

Physical health may also be affected by rumination. Cross-sectional data suggests rumination is related to reduced sleep quality [12,13,14] and less effective coping strategies, such as increased alcohol abuse [15]. Rumination has been linked to poorer cardiovascular health, by prolonging physiological reactions to stressors [4,16], and has been associated with increased cortisol and immune reactions following exposure to a stressor [17], although this may be influenced by context [18].

### 1.2. Restorative Environments

Restorative environments are spaces that offer the chance for physiological and psychological recovery from everyday stresses. They are typically conceptualized as pleasing, non-threatening, natural environments [19,20], but may also include man-made environments such as places of worship [21,22]. Natural environments appear to show restorative effects by increasing positive mood and reducing negative mood [23,24,25,26,27,28].

Exposure to natural, as opposed to urban, environments has been consistently associated with a range of psychological and physical benefits [3]. Beneficial effects have also been found in relation to simply viewing nature, or images of nature [3,26,27]. A systematic review found that self-reported emotions generally improved following activities in a natural environment, compared with the same activities in urban or laboratory settings [28]. Gardening may reduce depressive symptoms [29], whilst those recovering from surgery may be discharged sooner and take fewer analgesics if they have a view of nature, rather than buildings [30]. Walking and cycling appears to have a greater impact on general wellbeing when undertaken in green, rather than urban, environments [31]. Additionally, exposure to natural environments may also improve cognitive functioning [1,23] by reducing perceived levels of fatigue and improving attention. Following nature walks, as compared to urban walks, college students performed better on a proofreading task [24] and children with ADHD showed improved concentration [32]. Evidence for the effect of nature on cognitive functioning should be treated with caution, however, as meta-analyses have found limited evidence for such an effect [28,33].

### 1.3. Why Might Nature Benefit Rumination?

Two theoretical frameworks offer complementary explanations for the apparent restorative properties of nature; between them they suggest that exposure to nature enables restoration by improving mood and providing distraction. Stress Reduction Theory [34] is concerned with affective and physiological responses to threatening stimuli, and proposes that nature enables restoration via a reduction in arousal, with a resulting improvement in mood. Attention Restoration Theory [35,36] proposes that people become mentally fatigued in everyday life, and that nature enables cognitive restoration. Two proposed mechanisms involved in cognitive restoration, which are conceptualized as offering a form of distraction, are experiencing a sense of ‘being away’ from everyday concerns and a process called “soft fascination”, which describes how stimuli in the environment capture attention and provide a distraction [36].

Distraction and improving mood may be important mechanisms in reducing rumination, and these frameworks suggest that exposure to nature may provide these mechanisms. Rumination is positively correlated with negative mood and negatively correlated with positive mood [37] and experimentally inducing ruminative self-focus in young adults can lead to decreases in positive mood [38,39]. In contrast, spending time in nature is associated with the opposite effects on mood; exposure to nature increases positive mood and decreases negative mood [24,25,26,27]. Ruminative responses to depressive symptoms focus on an individual’s negative emotional state and maintain the depressed mood [5,6], whereas distracting responses that purposefully take the focus away from distress, such as engaging in neutral or pleasant activities, appear to offer relief from depressed mood [5].

Exposure to nature might therefore reduce rumination by providing a form of distraction and by improving mood. Successful interventions to reduce rumination include mindfulness [40] and distraction tasks [41,42]. Mindfulness interventions appear to increase positive mood and reduce rumination [43], whilst distraction reduces negative thinking [6]. A therapeutic horticulture intervention for individuals with depression, found that involvement with gardening activities significantly reduced levels of depressive mood and ruminative thinking [29]. Importantly, changes in mood were mediated by levels of being away and fascination [44]. The mechanisms by which these interventions appear to work suggest that exposure to nature might help individuals to disengage from rumination, by providing distraction from negative thoughts and by improving mood.

### 1.4. Hypotheses

Considering the two alternative mechanisms that might reduce rumination (improved mood and distraction), it was possible there would be an additive effect of nature, i.e. that rumination would be highest in the control condition, lowest in the nature condition, and somewhere in between in the urban condition. The experimental hypotheses were:

**Hypothesis** **1.** *There will be a greater reduction of task-related rumination in the nature condition, relative to the urban and control conditions*.

**Hypothesis** **2.** *There will be a difference in changes in positive mood (a greater increase) and in negative mood (a greater decrease) in the nature condition, relative to the urban and control conditions*.

**Hypothesis** **3.** *There will be higher levels of fascination and being away in the nature condition, relative to the urban and control conditions*.

## 2. Method

### 2.1. Participants

#### 2.1.1. Sample Size and Recruitment 

G*Power calculations [45] for a mixed ANOVA indicated a minimum sample size of 51 to find a large effect (*f* = 0.4) or 120 to find a medium effect (*f* = 0.25). For pragmatic reasons (primarily time and resource constraints), the target for recruitment was set at 60 (but with a minimum of 51); this was deemed appropriate as distraction has been shown to have a moderate to large effect on levels of rumination [46]. Additionally, exposure to nature has a moderate to large effect on mood [28]. 

Participants were recruited using hard-copy posters and flyers across the University campus. An electronic advert was also circulated on social media, via email and on the University’s online recruitment system. Of those who expressed an interest in participation (*n* = 94), 63 agreed to participate, and of those, 58 completed the study (five individuals cancelled participation bookings; no participants withdrew from the experiment). Participants were aged between 21 and 73 (*Median (Mdn)* = 27) and sample demographics are shown in Table 1.

Individuals were eligible to participate provided they were aged 18 years or older. No upper age limit was set, and there were no restrictions based on gender, ethnicity, or any other demographic variable. Participation was voluntary, and participants were advised they could withdraw without providing a reason. Written informed consent was taken from each participant. 

All participants who completed the study were offered entry into a prize draw to win one of two £25 shopping vouchers. To facilitate this, participants were asked to provide their email address on a separate form; this ensured email addresses were not connected to participant study data and could be stored separately. 

#### 2.1.2. Randomization Procedure

Participants were randomly allocated to one of three conditions: nature (*n* = 18), urban (*n* = 20), control (*n* = 20). Prior to study commencement, a set of random numbers were generated using an online random number generator [47]. The block randomization (without stratification) method was used, as this ensures equal numbers of participants across conditions, which is particularly important when sample sizes are relatively small [48]. Allocation was revealed as late as possible during the experiment to minimize any researcher bias and was concealed from participants. The randomization tool [47] was also used to enable the random presentation of items within measures to control for order effects. 

### 2.2. Materials and Measures

#### 2.2.1. Slideshows

**Figure 1:**
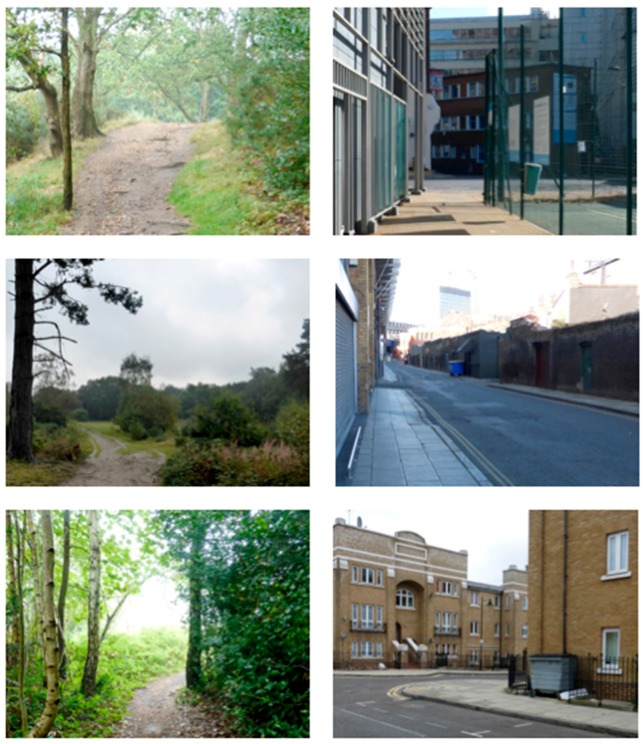
Sample images from natural and urban environment slideshows.

The slideshows were a series of still images, presented sequentially to represent a walk through either a natural environment (woodland and heathland in Southern England) or an urban environment (streets in London, England). Example images are reproduced in Figure 1. A research assistant at the University of Surrey created these slideshows from photographs taken between September 2014 and March 2015. No people were visible in either slideshow, which were matched for visual structure using a storyboard approach; for example, if the woodland path turned right, so did the road; if tall buildings framed an urban image, then tall trees framed the corresponding nature image. There was no soundtrack for either slideshow, as the perceived quality and content of soundscapes may interact with the restorative experience [49,50].

#### 2.2.2. Demographics

Participants’ age, gender, ethnicity, level of education, occupation, (coded as employed, retired, or student), and marital status were collected. Baseline measures of positive and negative mood, trait rumination, and connectedness-to-nature were also taken.

#### 2.2.3. Trait Rumination 

This was measured using the 22-item Ruminative Responses Scale which has three subscales measuring depression, reflective pondering and brooding, and has been shown to have good reliability [51]. Participants rated their tendency to think or behave in a certain way when they felt depressed using a Likert scale from one (almost never) to four (almost always).

#### 2.2.4. Connectedness-to-Nature 

This was measured using the 14-item “Connectedness-to-Nature” Scale, which is “designed to tap an individual’s affective, experiential connection to nature” [52] (p. 504) and has been shown to have high internal consistency and test-retest reliability [53]. Participants rated the extent to which they agreed with statements such as “I think of the natural world as a community to which I belong” using a Likert scale from one (strongly disagree) to five (strongly agree). 

#### 2.2.5. State Rumination 

This was measured using an adapted Thoughts Questionnaire [54] designed to capture ruminations about a presentation task, which has been used in similar studies of rumination [55,56]. The original questionnaire included items regarding feedback on a presentation, which formed part of the experimental procedure. As feedback was not a feature of the manipulation in the present study, these questions were omitted. The adapted 24-item version consisted of eight positive rumination statements (e.g., ‘how well I handled it’), fourteen negative rumination statements (e.g., ‘I made a fool of myself’), and two neutral statements, all focused on the presentation task. Participants rated how often they had experienced a given thought using a Likert scale from one (never) to five (very often). These scales have been shown to have good internal consistency, and are not highly correlated [54]. 

#### 2.2.6. Mood 

This was measured using the Positive and Negative Affect Schedule (PANAS) [57]. The PANAS consists of two 10-item mood scales and measures two distinct, although partially correlated, factors: positive affect and negative affect [58]. Participants rated to what extent they had experienced a given emotion using a Likert scale from one (very slightly or not at all) to five (extremely). The PANAS has been shown to have good reliability for both the positive and negative affect scales [57,58]. The effects of age, gender, education, and occupational status do not vary systematically across the PANAS [58], which has been utilized with different timeframes (e.g., “right now”, “over the last week/month”).

#### 2.2.7. Being Away and Fascination 

These were measured using the 5-item ‘Being Away’ and 8-item ‘Fascination’ subscales of the Perceived Restorativeness Scale [59]. These subscales have been shown to have acceptable reliability [60]. Participants rated their agreement with statements such as ‘being here is an escape experience’, or ‘this place is boring’, using a Likert scale from one (not at all) to seven (completely). Participants in the urban and nature conditions were asked to consider “the location you viewed in the video”; participants in the control condition were asked to consider “their surroundings”, i.e., the experimental room. 

#### 2.2.8. Reliability

Cronbach’s alphas were calculated for all measures and are reported in Table 2; for all but one subscale (in the baseline trait rumination scale), internal consistency was acceptable.

### 2.3. Procedure

#### 2.3.1. Overview

**Figure 2:**
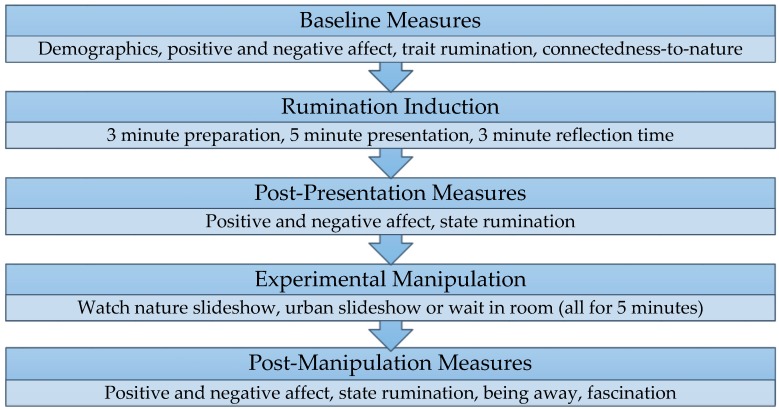
Summary of experimental procedure.

An experimental, within-between (Time x Condition) design was used to induce rumination in all participants, and then compare outcomes following exposure to different environments. For the experimental manipulation, participants either watched a slideshow of a natural or an urban environment, or waited in the room with no distractions. The slideshows were presented on a laptop. Dependent variables were changes over time in state rumination, positive mood, and negative mood. A between-participants design was also used to analyze post-manipulation differences in being away and fascination. Potential covariates were age [61], gender [13,62], trait rumination [4], and ‘connectedness-to-nature’ [53,63,64]. A summary of the experimental procedure is shown in Figure 2, and details of each step of the procedure are discussed below. The experiment was conducted by the first author. Data collection took place in a laboratory setting, at the University of Surrey, using paper questionnaires. Data was analyzed using IBM SPSS Statistics (Version 22, IBM Corp., Armonk, NY, USA).

#### 2.3.2. Baseline Procedure

Participants attended the experimental room individually, and sat at a corner table in the room. Throughout the study, the researcher was in the room with participants when instructions were given, but left the room whilst participants completed the measures and other steps in the procedure. 

First, participants provided demographic details, and completed baseline measures of positive mood, negative mood, trait rumination, and connectedness-to-nature. Participants were told they would be left alone to complete the questionnaires, but that the researcher would be in an adjoining room, behind a one-way mirror, where she could see and hear the participant at all times, who was instructed to indicate once they had completed the questionnaires. 

#### 2.3.3. Rumination Induction

Following this, a rumination induction took place. This consisted of a presentation task, followed by a period of reflection; a similar task has been used in previous studies of rumination [55,56]. Participants were asked to imagine they had come for a job interview, and were advised they would be given three minutes to prepare for a five-minute presentation, the purpose of which was to explain why they were the best candidate for the job. The job was not specified; participants were told they were free to choose the imagined vacancy, based on their own job history. They were advised that when the preparation time was up, they would need to stand in the center of the room, at a lectern, and present to a one-way mirror, behind which an interview panel would be observing them. Participants were advised they did not need to keep track of time, as the researcher would prompt them when their preparation time and their presentation time was over. Participants were provided with paper to make notes during the preparation, and given the opportunity to ask clarifying questions. Participants were advised that after their presentation they would be given feedback on their performance. 

After the preparation time, the researcher used an intercom system to prompt participants to begin their presentation. If the participant stopped speaking for more than a few seconds, they were prompted to continue, with questions such as “please tell me a bit more about that project/role”. During the presentation, the researcher took notes to form the basis of the feedback. After five minutes had passed, participants were informed that their time was up and were instructed to “reflect on your performance for a short time, and think about how you did with your speech” (they were left to reflect for three minutes, but were not told how long this period would be). These instructions were designed to elicit rumination on the presentation task. 

#### 2.3.4. Post-Presentation Procedure

Following the reflection period, participants completed post-presentation measures of positive mood, negative mood, and state rumination (this also served as a mood manipulation check). At this point, the researcher opened the relevant condition allocation envelope.

#### 2.3.5. Experimental Manipulation

Once post-presentation measures had been completed, participants were advised their feedback would now be prepared. Control participants were asked to wait patiently in the room for a few minutes, and were instructed not to check any mobile devices. Participants allocated to the nature and urban conditions were advised they would watch a short video (the slideshow) whilst they waited. Each slideshow lasted five minutes; control participants were left alone for the same length of time.

#### 2.3.6. Post-Manipulation Procedure

Next, participants completed the rumination and mood measures again, along with measures of being away and fascination. The researcher then returned to the room and provided feedback on the presentation. Participants were thanked for their time and fully debriefed, and were offered the opportunity to enter the prize draw. The whole procedure lasted around 45 min.

### 2.4. Ethics

Favorable ethical opinion was granted from the Faculty of Arts and Human Sciences Ethics Committee at the University of Surrey (project code: 1121-PSY-15), and the study was conducted in accordance with the Declaration of Helsinki. As the study involved a negative mood manipulation (the rumination induction), a positive mood manipulation was included during participant debriefing, in the form of appropriate, positive feedback on the participant’s presentation. The researcher ensured participants were not distressed before they left the study location. Participants were verbally debriefed about the study’s purpose and given a debrief sheet detailing a study summary and contact details for the research team. Please note that research data cannot be made publicly available in a data repository as participants were not consented for this. 

## 3. Results

### 3.1. Missing Data

No cases had missing values for the units of analysis (total scores on each measure). Five (8.62%) cases had missing data at the level of individual items on various scales. Of all possible item values, 0.06% (6/9506 items) were missing, and were deemed to be missing completely at random; this was confirmed by performing Little’s Missing Completely At Random Test *χ*^2^ = 1.42, *p* = 1.0. Therefore, values for missing items were not calculated, and all cases were included in the final analysis. 

### 3.2. Distribution and Baseline Checks

All continuous variables across all time-points and conditions were normally distributed except for age and negative mood (at post-presentation and post-manipulation). Baseline differences in age were assessed using a Kruskal-Wallis test; baseline differences for all other continuous variables were assessed using one-way independent ANOVAs. Cell frequencies were examined for categorical demographic variables; in all instances, minimum cell frequencies were not met, so Fisher’s exact test was used to test for baseline differences in categorical variables. There were no significant differences between conditions at baseline on any variable (see Table 3). Demographic details and descriptive statistics for baseline variables are reported in Appendix A (see Table A1).

### 3.3. Mood Manipulation Check

The effect of the presentation task on positive mood was investigated using a repeated-measures *t*-test. As expected, positive mood across the sample reduced from baseline (*M* = 34.53, *SD* = 6.42) to post-presentation (*M* = 28.41, *SD* = 7.59). This difference, −6.12, 95% CI [−3.93, −8.31], was significant and represented a large effect size, *t*(57) = 5.60, *p* < 0.001, *d* = 0.87. 

The effect of the presentation task on negative mood was also tested, using a Wilcoxon signed-rank test. Negative mood reduced across the sample from baseline (*Mdn* = 17, *Range* = 21) to post-presentation (*Mdn* = 15, *Range* = 31). This difference, −2.00, was also significant and represented a small effect size, *T* = 494, *p* = 0.033, *r* = 0.20, but was in the opposite direction to expectations. 

The presentation task therefore significantly reduced both positive and negative mood. To check these effects were consistent across conditions, post-presentation differences for positive mood and state rumination were assessed using one-way independent ANOVAs, whilst a Kruskal-Wallis test was used for post-presentation negative mood. There were no significant differences between groups (see Table 4). Descriptive statistics are reported in Appendix B (see Table A2).

### 3.4. Main Findings

As there were no group differences in any variables at either baseline or post-presentation, no covariates were controlled for in any of the final analyses. Descriptive statistics for all post-manipulation scores are reported in Table 5.

#### 3.4.1. State Rumination

**Figure 3:**
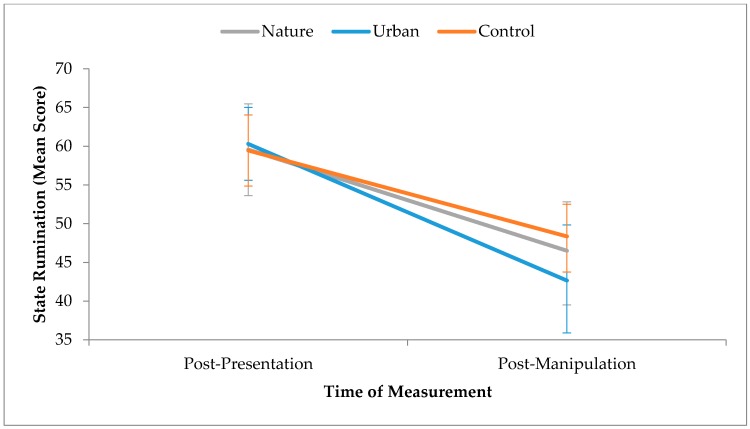
Effect of time on state rumination by condition. Error bars represent 95% confidence intervals.

Changes in state rumination were tested using a mixed (Time x Condition) ANOVA. There was no significant main effect of environment on changes in state rumination, *F*(2,55) = 0.27, *p* = 0.77. There was, however, a significant main effect of time on changes in state rumination, *F*(1,55) = 71.00, *p* < 0.001, partial *η*^2^ = 0.56, which represents a large effect size; state rumination significantly reduced from post-presentation to post-manipulation across all three conditions (see Figure 3). There was no significant Time x Condition interaction, *F*(2,55) = 1.42, *p* = 0.25. Therefore, although state rumination reduced in all three conditions over time, exposure to different environments did not influence the level of reduction.

#### 3.4.2. Positive Mood

**Figure 4:**
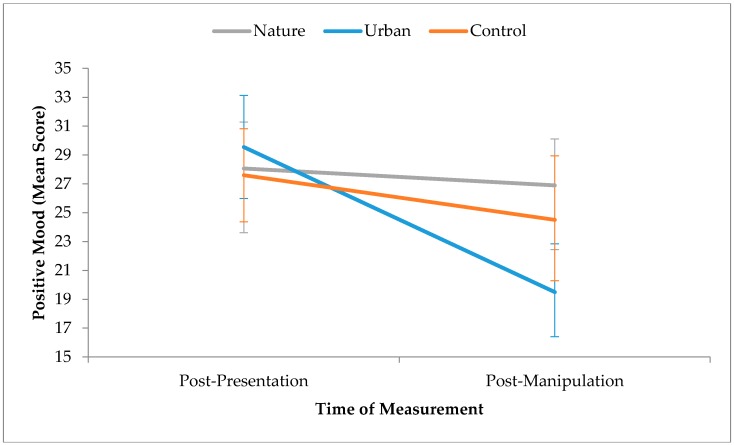
Time x Condition interaction effects for positive mood. Error bars represent 95% confidence intervals.

To test whether positive mood changed across time as a function of condition, a mixed (Time x Condition) ANOVA was performed. This revealed a significant Time x Condition interaction for positive mood, *F*(2,55) = 11.83, *p* < 0.001, partial *η*^2^ = 0.30 (see Figure 4), which was explored using planned comparisons. 

Simple effects analysis showed no significant difference between conditions on positive mood at post-presentation, *F*(2,55) = 0.35, *p* = 0.71, but there was a significant difference between conditions at post-manipulation, *F*(2,55) = 3.55, *p* = 0.035, partial *η*^2^ = 0.11. Contrasts revealed the effect of time was significant in the urban, *F*(1,55) = 56.50, *p* < 0.001, partial *η*^2^ = 0.51, and control conditions, *F*(1,55) = 5.37, *p* = 0.024, partial *η*^2^ = 0.08, but not the nature condition, *F*(1,55) = 0.68, *p* = 0.41, partial *η*^2^ = 0.01. Environmental exposure therefore significantly influenced changes in positive mood over time. Participants in the urban condition reported a large and significant decrease in positive mood, whilst participants in the control condition reported a medium and significant decrease. Participants in the nature condition, however, reported only a small decrease in positive mood, and the effect was non-significant. 

#### 3.4.3. Negative Mood 

Because post-presentation and post-manipulation negative mood were positively skewed, a mixed ANOVA could not be conducted. Instead, a new variable was computed to calculate the difference between post-presentation and post-manipulation negative mood scores. This was deemed acceptable, as there were no differences between groups on negative mood at either baseline or post-presentation. This new variable was also positively skewed, so a Kruskal-Wallis test was performed to test for differences between conditions in changes in negative mood. The results showed no significant effect of environment on changes in negative mood, *H*(2) = 2.14, *p* = 0.34. 

**Figure 5:**
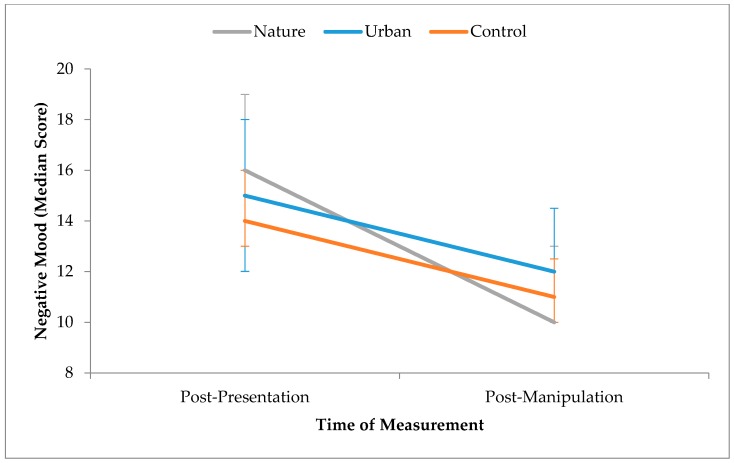
Effect of time by condition for negative mood. Error bars represent 95% confidence intervals.

A Wilcoxon signed-rank test was performed to investigate whether negative mood changed as a function of time across the sample. Negative mood reduced from post-presentation (*Mdn* = 15.00, *Range* = 31.00) to post-manipulation (*Mdn* = 11.00, *Range* = 22.00). This difference, −4.00, was significant, *T* = 88.50, *p* < 0.001, *r* = 0.50, and represented a large effect size. Therefore, although negative mood did significantly reduce between post-presentation and post-manipulation, these changes were not affected by exposure to different environments (see Figure 5). 

#### 3.4.4. Fascination 

**Figure 6:**
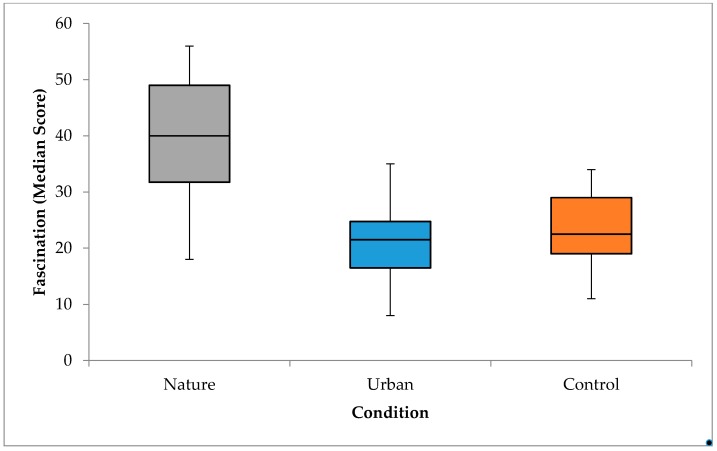
Differences in fascination between conditions at post-manipulation. Upper limits for urban and control conditions do not include outliers (urban = 37, 44; control = 47); there were no outliers for the nature condition.

A one-way independent ANOVA was used to test whether fascination differed between conditions. Fascination was significantly affected by environment, *F*(2,55) = 17.82, *p* < 0.001, partial *η*^2^ = 0.39 (see Figure 6). Post hoc tests (Tukey) showed that fascination was significantly higher in the nature condition compared to both the urban, *p* < 0.001, *d* = 1.65, and control conditions, *p* < 0.001, *d* = 1.58, which represent large effects. There was no significant difference in fascination between the urban and control conditions, *p* = 0.94. Participants perceived the nature slideshow to be significantly more fascinating than the experimental room or the urban slideshow. 

#### 3.4.5. Being Away 

**Figure 7:**
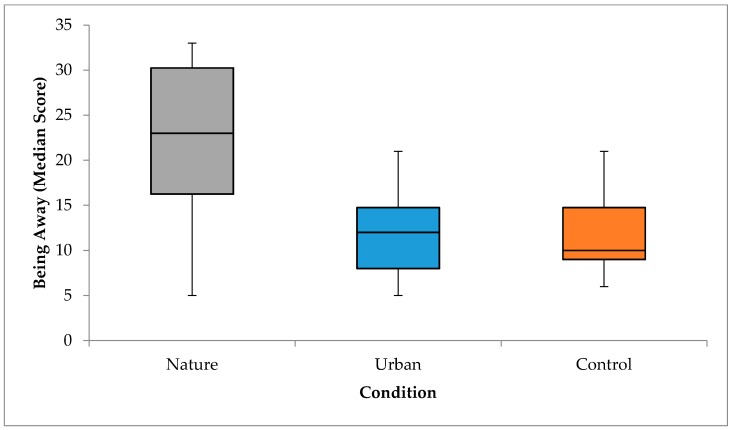
Differences in being away between conditions at post-manipulation.

A one-way independent ANOVA was used to test whether being away differed between conditions. Being away was significantly affected by environment, *F*(2,55) = 20.91, *p* < 0.001, partial *η*^2^ = 0.43 (see Figure 7). Post hoc tests (Tukey) showed that being away was significantly higher in the nature condition compared to both the urban, *p* < 0.001, *d* = 1.63 and control conditions, *p* < 0.001, *d* = 1.79, which are both large effects. There was no significant difference in being away between the urban and control conditions, *p* = 0.97. Participants exposed to the nature slideshow reported a significantly greater sense of being away compared to those who watched the urban slideshow, or who waited in the room. 

## 4. Discussion

This study explored the relationship between restorative environments and rumination. Specifically, it investigated the effects of exposure to photographic slideshows of different environments on levels of task-related rumination, following a presentation task designed to induce rumination. It was hypothesized that participants who watched a slideshow of a walk through a natural environment would report a greater reduction in rumination than participants who watched a slideshow of a walk through an urban environment or experienced the control condition. It was also hypothesized that participants who watched the slideshow of the nature walk would have higher positive mood, lower negative mood, and higher levels of being away and fascination compared to participants in the urban and control conditions. 

The results of this study did not support the main hypothesis (Hypothesis 1); reductions in task-related rumination were not influenced by environmental exposure. Participants did report a reduction in rumination from immediately after the presentation to immediately after the slideshow (or control period of waiting), which suggests that there was an effect of the rumination induction on state rumination, but there was no significant difference between conditions. Rumination therefore decreased over time, but was not influenced by environmental exposure. This outcome may be related to the fact that negative mood decreased following the presentation task. It was expected that, compared to baseline, positive mood would reduce and negative mood would increase following the presentation. Whilst positive mood did indeed change as expected after the presentation (it decreased), negative mood did not; instead, negative mood also decreased following the presentation.

Rumination is associated with negative mood, and it is thought that negative emotional engagement with ruminative thoughts contributes to the persistent nature of rumination [6,40,65]. Participants did report thinking about their presentation immediately after they gave their speech (after all, they were instructed to do so). However, the reduced levels of negative mood after the presentation, as compared to baseline, suggest participants were perhaps not sufficiently emotionally engaged with the presentation task. This lack of a negative emotional engagement with their thoughts about their presentation may have contributed to ruminative thinking not being maintained, which may be one reason why there was no difference in rumination across conditions.

Another possible reason for a lack of an observed effect on rumination is that participants in this sample were simply not prone to ruminating. Many studies investigating rumination select for participants with high trait rumination or who are depressed [37,42,43]. Nonetheless, other researchers have successfully induced rumination in participants recruited from the general population [39,46], so it is still considered appropriate to have recruited from the general population for this study. 

Hypothesis 2 was partially supported. There were large and significant differences in positive mood over time between the three conditions, although there were no differences in negative mood. As expected, positive mood decreased following the presentation task. Following the experimental manipulation there were further changes to positive mood, with post-manipulation scores being highest amongst those who watched the nature slideshow. Interestingly though, positive mood did not recover to baseline levels; this contrasts with previous studies, which have shown that exposure to nature facilitates a recovery to baseline levels of positive mood, following a dip immediately after a stressor task (e.g., [23]). In the present study, rather than facilitating recovery in positive mood back to baseline levels, exposure to nature seems to have merely prevented a further dip in positive mood that was seen in the urban and control conditions. Although there was a significant difference between all three conditions, the confidence intervals and effect sizes for mean post-manipulation positive mood indicate that the largest and most meaningful difference was between the nature and urban conditions, with a smaller difference between nature and control participants. This finding supports and adds to existing research that suggests natural and urban environments have differing effects on positive mood.

Despite these promising results for positive mood, there was not a corresponding influence of environment on negative mood. As with state rumination, negative mood decreased significantly from post-presentation to post-manipulation, but the size of the reduction was not affected by environmental exposure. Although some studies have found that negative mood is influenced by environment [2,25], this is not a universal finding. The results of this study are in line with another study that found that although negative mood improved over the short-term, this was not related to environmental exposure [23]. Despite relatively consistent evidence for the influence of nature on positive mood, the effects of nature on negative mood remain unclear.

Finally, Hypothesis 3 was supported. Participants who watched the nature slideshow reported significantly higher levels of being away and fascination than did control participants or those who watched the urban slideshow. Analysis revealed these differences represented large effect sizes. These findings are in line with previous research that has demonstrated that exposure to natural environments elicits greater levels of being away and fascination than does exposure to urban environments [44].

### Limitations

Possible limitations in the measurement of negative mood in this study should be acknowledged, and these observations should be improved upon in future research. It may be that the lack of a negative emotional engagement is an artefact of the measure used. Although the PANAS [57] is often used in restoration research and has repeatedly detected changes to both positive and negative mood [23,25], it was perhaps not fully appropriate for the rumination induction used in this study. Participants were asked to rate how they felt “right now” following the presentation and reflection period, yet several participants reported during debriefing that despite being stressed during the task, they then simply felt relief at having completed the presentation. 

Potentially, the control condition may not have been fully effective as a ‘non-distraction’ situation, although it is difficult to conceive of a suitable alternative control activity. Some participants in the control condition reported that their mind wandered or that they actively distracted themselves (e.g., by planning their day, or counting chairs in the room), which may have impacted upon rumination in the control group. Intriguingly, the distraction effects of both slideshows may be over-estimated; some participants reported paying close attention to the slideshows as they were expecting to be asked questions about what they had seen. This suggests that, at least for some participants, the slideshows were distracting (leading to a reduction in levels of state rumination), but for the wrong reason (i.e., participants had expectations about being in an experiment). Nonetheless, large effects of environmental exposure were seen for positive mood, being away, and fascination.

A further limitation is related to the relatively small sample size. Although the study was sufficiently powered to explore the main hypotheses, a larger sample would have enabled an examination of individual differences in relation to trait rumination and connectedness-to-nature. A larger sample size would also have enabled exploration of any potential differences between conditions according to whether participants scored highly on the positive and negative rumination subscales of the state rumination measure. Future research could aim to explore whether such individual differences might be related to differences in levels of state rumination immediately after a rumination induction and after exposure to different environments. 

Despite these limitations, steps were taken to minimize bias in the study, and the random allocation appears to have been successful, as indicated by a lack of baseline differences between participants across conditions. Encouragingly, participants who watched the nature slideshow reported significantly greater levels of being away and fascination than did those who watched the urban slideshow or waited in the room. In previous restorative environment studies, these constructs have consistently been rated higher amongst participants who experienced natural environments, as compared to those who experienced urban environments [25,66]. It therefore seems reasonable to conclude that the slideshows used in this study were good representations of natural and urban environments for the purposes of exploring restorative experiences. The lack of an observed effect of environment upon rumination is therefore unlikely to be attributable to the quality of the stimuli. 

## 5. Conclusions

The findings from this study contribute to knowledge of restorative environments in three key ways. First, to the best of our knowledge, this study is unique in having specifically explored the relationship between images of restorative environments and rumination. Although the nature slideshow was rated more fascinating (distracting) than the urban slideshow or experimental room, in this study nature was no more or less effective at distracting individuals from task-related ruminative thinking. Future research could explore any effects nature may have upon other, more persistent forms of rumination, such as depressive rumination or work-related rumination in those with high job-strain. Second, the encouraging findings in relation to positive mood, being away, and fascination suggest the slideshows used in this study are a valid representation of restorative and non-restorative environments, and could therefore be utilized for further research. Finally, this study adds to existing evidence that natural and urban environments have different effects on positive mood, whilst also highlighting the need for further research to clarify exactly how different environments may influence negative mood.

## Figures and Tables

**Table 1:** Demographic Details for Whole Sample and by Condition.

Characteristic	Individuals in Sample *n* = 58	Percentage of Sample	Individuals in Nature Condition *n* = 18	Individuals in Built Condition *n* = 20	Individuals in Control Condition *n* = 20
Gender					
Female	45	77.6	14	15	16
Male	13	22.4	4	5	4
Ethnicity *					
White British	30	51.7	9	9	12
Indian British	2	3.4	1	0	1
Mixed	4	6.9	1	2	1
White Other	19	32.8	5	8	6
Asian Other	1	1.7	1	0	0
Other	2	3.4	1	1	0
Education Level					
No qualifications	1	1.7	1	0	0
GCSE/O-Level	5	8.6	1	2	2
A-Level/AS-Level	4	6.9	0	3	1
Diploma/HND	3	5.2	1	0	2
Degree	18	31.0	5	7	6
Postgraduate degree/diploma	27	46.6	10	8	9
Marital Status *					
Single	17	29.3	4	9	4
Dating	16	27.6	5	6	5
Co-habiting	5	8.6	1	3	1
Married/Civil Partnership	18	31.0	8	2	8
Divorced/Separated	1	1.7	0	0	1
Widowed	1	1.7	0	0	1
Employment Status					
Student	27	46.6	6	12	9
Employed	30	51.7	12	8	10
Retired	1	1.7	0	0	1

* Percentages do not exactly total 100% due to rounding.

**Table 2:** Reliability Statistics for all Continuous Measures.

Measure	Time		
	Baseline	Post-Presentation	Post-Manipulation
	Cronbach’s α	Cronbach’s α	Cronbach’s α
Connectedness-to-nature	0.80		
Trait rumination—depression subscale	0.84		
Trait rumination—brooding subscale	0.58		
Trait rumination—reflective pondering subscale	0.72		
Positive affect	0.87	0.90	0.95
Negative affect	0.81	0.88	0.87
State rumination—positive thoughts subscale		0.81	0.91
State rumination—negative thoughts subscale		0.89	0.94
Being away			0.89
Fascination			0.92

**Table 3:** Significance Tests for Baseline Differences Across all Variables.

Measure	Fisher’s Exact Test	*F*-Ratio (df)	*H* (df)	*p*-Value
Gender	0.24			1.00
Ethnicity	6.17			0.92
Education	7.59			0.73
Marital status	12.1			0.19
Employment	4.45			0.27
Age			2.93 (2)	0.23
Positive affect		1.37 (2, 55)		0.26
Negative affect		2.05 (2, 55)		0.14
Connectedness-to-nature		0.13 (2, 55)		0.88
Trait rumination		0.26 (2, 55)		0.78

Notes: df = degrees of freedom, *H* = Kruskal-Wallis test statistic.

**Table 4:** Significance Tests for Post-Presentation Differences.

Measure	*F*-Ratio (df)	*H* (df)	*p*-Value
Positive affect	0.35 (2, 55)		0.71
Negative affect		0.37 (2)	0.83
State rumination	0.04 (2, 55)		0.96

Notes: df = degrees of freedom, *H* = Kruskal-Wallis test statistic.

**Table 5:** Descriptive Statistics at Post-Manipulation by Condition.

Measure	Nature		Built		Control	
	*M/Mdn* (95% CI)	*SD/Range*	*M/Mdn* (95% CI)	*SD/Range*	*M/Mdn* (95% CI)	*SD/Range*
Positive affect	26.89 (22.67, 31.33)	9.57	19.50 (16.40, 22.85)	7.42	24.50 (20.60, 28.20)	9.27
Negative affect *	10.00 (10.00, 13.00)	9.00	12.00 (10.00, 14.50)	11.00	11.00 (10.00, 12.50)	22.00
Negative affect difference (computed variable) *	5.00 (1.50, 7.00)	31.00	2.00 (1.00, 5.00)	16.00	3.00 (1.00, 4.00)	14.00
State rumination	46.50 (39.50, 52.83)	14.84	42.65 (35.90, 49.85)	15.96	48.35 (43.75, 52.50)	10.32
Being Away	22.33 (18.42, 26.25)	7.87	11.95 (9.69, 14.21)	4.84	11.50 (9.51, 13.49)	4.25
Fascination	39.44 (33.69, 45.20)	11.57	22.60 (18.44, 26.76)	8.89	23.65 (19.72, 27.58)	8.41

Notes: 95% CI = bootstrapped 95% confidence interval. *M* = mean. *Mdn* = median. *SD* = standard deviation. * Median and range reported for negative affect.

## Appendix A

**Table A1:** Descriptive Statistics and Results of Tests of Differences at Baseline by Condition.

Measure	Nature		Built		Control			
	*M/Mdn* (95% CI)	*SD/Range*	*M/Mdn* (95% CI)	*SD/Range*	*M/Mdn* (95% CI)	*SD/Range*	*F*-Ratio/*H* (df)	*p*-Value
Age *	27.00 (24.00, 37.50)	50	25.50 (23.00, 28.00)	29	28.50 (24.00, 41.00)	45	2.93 (2)	0.23
Positive affect	32.50 (29.04, 35.96)	6.96	35.75 (32.55, 38.95)	6.84	35.15 (32.68, 37.62)	5.27	1.37 (2, 55)	0.26
Negative affect	17.11 (14.02, 20.20)	6.22	20.75 (17.74, 23.76)	6.43	17.70 (15.19, 20.21)	5.37	2.05 (2, 55)	0.14
Connectedness-to-nature	48.89 (45.34, 52.44)	7.13	49.00 (45.41, 52.59)	7.66	50.00 (46.43, 53.57)	7.64	0.13 (2, 55)	0.88
Trait rumination	47.11 (41.38, 52.84)	11.52	49.20 (44.20, 54.20)	10.68	47.00 (42.08, 51.92)	10.52	0.26 (2, 55)	0.78

Notes: 95% CI = bootstrapped 95% confidence interval. *M* = mean. *Mdn* = median. *SD* = standard deviation. df = degrees of freedom. * Median, range and Kruskal-Wallis statistic reported for Age.

## Appendix B

**Table A2:** Descriptive Statistics and Results of Tests of Differences at Post-Presentation by Condition.

Measure	Nature		Built		Control			
	*M/Mdn* (95% CI)	*SD/Range*	*M/Mdn* (95% CI)	*SD/Range*	*M/Mdn* (95% CI)	*SD/Range*	*F*-Ratio/*H* (df)	*p*-Value
Positive affect	28.06 (24.83, 31.28)	6.49	29.55 (25.98, 33.12)	7.62	27.60 (23.55, 31.65)	8.65	0.35 (2, 55)	0.71
Negative affect *	16.00 (12.00, 18.99)	31	15.00 (12.01, 18.00)	23	14.00 (13.00, 16.00)	24	0.37 (2)	0.83
State rumination	59.56 (53.63, 65.48)	11.92	60.30 (55.59, 65.01)	10.07	59.45 (54.86, 64.04)	9.80	0.04 (2, 55)	0.96

Notes: 95% CI = bootstrapped 95% confidence interval. *M* = mean. *Mdn* = median. *SD* = standard deviation. df = degrees of freedom. * Median, range and Kruskal-Wallis statistic reported for negative affect.

## References

[1] Berman M.G., Jonides J., Kaplan S. (2008). The cognitive benefits of interacting with nature. Psychol. Sci..

[2] Hartig T., Evans G.W., Jamner L.D., Davis D.S., Gärling T. (2003). Tracking restoration in natural and urban field settings. J. Environ. Psychol..

[3] Velarde M.D., Fry G., Tveit M. (2007). Health effects of viewing landscapes—Landscape types in environmental psychology. Urban For. Urban Green..

[4] Brosschot J.F., Gerin W., Thayer J.F. (2006). The perseverative cognition hypothesis: A review of worry, prolonged stress-related physiological activation, and health. J. Psychosom. Res..

[5] Nolen-Hoeksema S. (1991). Responses to depression and their effects on the duration of depressive episodes. J. Abnorm. Psychol..

[6] Nolen-Hoeksema S., Wisco B.E., Lyubomirsky S. (2008). Rethinking rumination. Perspect. Psychol. Sci..

[7] Mor N., Winquist J. (2002). Self-focused attention and negative affect: A meta-analysis. Psychol. Bull..

[8] Muris P., Roelofs J., Rassin E., Franken I., Mayer B. (2005). Mediating effects of rumination and worry on the links between neuroticism, anxiety and depression. Personal. Individ. Differ..

[9] Nolen-Hoeksema S. (2000). The role of rumination in depressive disorders and mixed anxiety/depressive symptoms. J. Abnorm. Psychol..

[10] Cropley M., Millward Purvis L. (2003). Job strain and rumination about work issues during leisure time: A diary study. Eur. J. Work Organ. Psychol..

[11] Cropley M., Zijlstra F.R.H., Langan-Fox J., Cooper C.L. (2011). Work and rumination. Handbook of Stress in the Occupations.

[12] Berset M., Elfering A., Lüthy S., Lüthi S., Semmer N.K. (2011). Work stressors and impaired sleep: Rumination as a mediator. Stress Health.

[13] Cropley M., Dijk D.-J., Stanley N. (2006). Job strain, work rumination, and sleep in school teachers. Eur. J. Work Organ. Psychol..

[14] Querstret D., Cropley M. (2012). Exploring the relationship between work-related rumination, sleep quality, and work-related fatigue. J. Occup. Health Psychol..

[15] Nolen-Hoeksema S., Harrell Z.A. (2002). Rumination, depression, and alcohol use: Tests of gender differences. J. Cogn. Psychother..

[16] Key B.L., Campbell T.S., Bacon S.L., Gerin W. (2008). The influence of trait and state rumination on cardiovascular recovery from a negative emotional stressor. J. Behav. Med..

[17] Zoccola P.M., Figueroa W.S., Rabideau E.M., Woody A., Benencia F. (2014). Differential effects of poststressor rumination and distraction on cortisol and C-reactive protein. Health Psychol..

[18] Zoccola P.M., Dickerson S.S. (2012). Assessing the relationship between rumination and cortisol: A review. J. Psychosom. Res..

[19] Andrews M., Gatersleben B. (2010). Variations in perceptions of danger, fear and preference in a simulated natural environment. J. Environ. Psychol..

[20] Gatersleben B., Andrews M. (2013). When walking in nature is not restorative—The role of prospect and refuge. Health Place.

[21] Herzog T.R., Ouellette P., Rolens J.R., Koenigs A.M. (2009). Houses of worship as restorative environments. Environ. Behav..

[22] Ouellette P., Kaplan R., Kaplan S. (2005). The monastery as a restorative environment. J. Environ. Psychol..

[23] Berman M.G., Kross E., Krpan K.M., Askren M.K., Burson A., Deldin P.J., Kaplan S., Sherdell L., Gotlib I.H., Jonides J. (2012). Interacting with nature improves cognition and affect for individuals with depression. J. Affect. Disord..

[24] Hartig T., Mang M., Evans G.W. (1991). Restorative effects of natural environment experiences. Environ. Behav..

[25] Tyrväinen L., Ojala A., Korpela K., Lanki T., Tsunetsugu Y., Kagawa T. (2014). The influence of urban green environments on stress relief measures: A field experiment. J. Environ. Psychol..

[26] Ulrich R.S., Simons R.F., Losito B.D., Fiorito E., Miles M.A., Zelson M. (1991). Stress recovery during exposure to natural and urban environments. J. Environ. Psychol..

[27] Van den Berg A.E., Koole S.L., van der Wulp N.Y. (2003). Environmental preference and restoration: (How) are they related?. J. Environ. Psychol..

[28] Bowler D.E., Buyung-Ali L.M., Knight T.M., Pullin A.S. (2010). A systematic review of evidence for the added benefits to health of exposure to natural environments. BMC Public Health.

[29] Gonzalez M.T., Hartig T., Patil G.G., Martinsen E.W., Kirkevold M. (2009). Therapeutic horticulture in clinical depression: A prospective study. Res. Theory Nurs. Pract..

[30] Ulrich R.S. (1984). View through a window may influence recovery from surgery. Science.

[31] Pretty J., Peacock J., Hine R., Sellens M., South N., Griffin M. (2007). Green exercise in the UK countryside: Effects on health and psychological well-being, and implications for policy and planning. J. Environ. Plan. Manag..

[32] Taylor A.F., Kuo F.E. (2009). Children with attention deficits concentrate better after walk in the park. J. Atten. Disord..

[33] Ohly H., White M.P., Wheeler B.W., Bethel A., Ukoumunne O.C., Nikolaou V., Garside R. (2016). Attention restoration theory: A systematic review of the attention restoration potential of exposure to natural environments. J. Toxicol. Environ. Health Part B.

[34] Ulrich R.S., Altman I., Wohlwill J.F. (1983). Aesthetic and affective response to natural environment. Behavior and the Natural Environment.

[35] Kaplan S., Kaplan R. (1989). The restorative environment. The Experience of Nature: A Psychological Perspective.

[36] Kaplan S. (1995). The restorative benefits of nature: Toward an integrative framework. J. Environ. Psychol..

[37] McLaughlin K.A., Borkovec T.D., Sibrava N.J. (2007). The effects of worry and rumination on affect states and cognitive activity. Behav. Ther..

[38] Huffziger S., Ebner-Priemer U., Eisenbach C., Koudela S., Reinhard I., Zamoscik V., Kirsch P., Kuehner C. (2013). Induced ruminative and mindful attention in everyday life: An experimental ambulatory assessment study. J. Behav. Ther. Exp. Psychiatry.

[39] Huffziger S., Ebner-Priemer U., Koudela S., Reinhard I., Kuehner C. (2012). Induced rumination in everyday life: Advancing research approaches to study rumination. Personal. Individ. Differ..

[40] Querstret D., Cropley M. (2013). Assessing treatments used to reduce rumination and/or worry: A systematic review. Clin. Psychol. Rev..

[41] Kuehner C., Huffziger S., Liebsch K. (2009). Rumination, distraction and mindful self-focus: Effects on mood, dysfunctional attitudes and cortisol stress response. Psychol. Med..

[42] Park R.J., Goodyer I.M., Teasdale J.D. (2004). Effects of induced rumination and distraction on mood and overgeneral autobiographical memory in adolescent Major Depressive Disorder and controls. J. Child Psychol. Psychiatry.

[43] Huffziger S., Kuehner C. (2009). Rumination, distraction, and mindful self-focus in depressed patients. Behav. Res. Ther..

[44] Gonzalez M.T., Hartig T., Patil G.G., Martinsen E.W., Kirkevold M. (2010). Therapeutic horticulture in clinical depression: A prospective study of active components. J. Adv. Nurs..

[45] Faul F., Erdfelder E., Lang A.G., Buchner A. (2007). G*Power 3: A flexible statistical power analysis program for the social, behavioral, and biomedical sciences. Behav. Res. Methods.

[46] Rood L., Roelofs J., Bögels S.M., Arntz A. (2012). The effects of experimentally induced rumination, positive reappraisal, acceptance, and distancing when thinking about a stressful event on affect states in adolescents. J. Abnorm. Child Psychol..

[47] Urbaniak G.C., Plous S. (2015). Research Randomizer (Version 4.0). http://www.randomizer.org/.

[48] Kim J., Shin W. (2014). How to do random allocation (randomization). Clin. Orthop. Surg..

[49] Alvarsson J.J., Wiens S., Nilsson M.E. (2010). Stress recovery during exposure to nature sound and environmental noise. Int. J. Environ. Res. Public Health.

[50] Ratcliffe E., Gatersleben B., Sowden P.T. (2013). Bird sounds and their contributions to perceived attention restoration and stress recovery. J. Environ. Psychol..

[51] Treynor W., Gonzalez R., Nolen-Hoeksema S. (2003). Rumination reconsidered: A psychometric analysis. Cogn. Ther. Res..

[52] Mayer F.S., Frantz C.M. (2004). The connectedness to nature scale: A measure of individuals’ feeling in community with nature. J. Environ. Psychol..

[53] Mayer F.S., Frantz C.M., Bruehlman-Senecal E., Dolliver K. (2008). Why is nature beneficial? The role of connectedness to nature. Environ. Behav..

[54] Edwards S.L., Rapee R.M., Franklin J. (2003). Postevent rumination and recall bias for a social performance event in high and low socially anxious individuals. Cogn. Ther. Res..

[55] Zoccola P.M., Dickerson S.S., Zaldivar F.P. (2008). Rumination and cortisol responses to laboratory stressors. Psychosom. Med..

[56] Zoccola P.M., Dickerson S.S., Lam S. (2012). Eliciting and maintaining ruminative thought: The role of social-evaluative threat. Emotion.

[57] Watson D., Clark L.A., Tellegen A. (1988). Development and validation of brief measures of positive and negative affect: The PANAS scales. J. Personal. Soc. Psychol..

[58] Crawford J.R., Henry J.D. (2004). The Positive and Negative Affect Schedule (PANAS): Construct validity, measurement properties and normative data in a large non-clinical sample. Br. J. Clin. Psychol..

[59] Hartig T., Korpela K., Evans G.W., Gärling T. (1997). A measure of restorative quality in environments. Scand. Hous. Plan. Res..

[60] Laumann K., Gärling T., Stormark K.M. (2001). Rating scale measures of restorative components of environments. J. Environ. Psychol..

[61] Sütterlin S., Paap M.C.S., Babic S., Kübler A., Vögele C. (2012). Rumination and age: Some things get better. J. Aging Res..

[62] Nolen-Hoeksema S., Jackson B. (2001). Mediators of the gender difference in rumination. Psychol. Women Q..

[63] Koole S.L., van den Berg A.E. (2005). Lost in the wilderness: Terror management, action orientation, and nature evaluation. J. Personal. Soc. Psychol..

[64] Davis N., Gatersleben B. (2013). Transcendent experiences in wild and manicured settings: The influence of the trait “Connectedness to Nature”. Ecopsychology.

[65] Watkins E.R. (2008). Constructive and unconstructive repetitive thought. Psychol. Bull..

[66] Herzog T.R., Maguire P., Nebel M.B. (2003). Assessing the restorative components of environments. J. Environ. Psychol..

